# Conservative Management of Placenta Accreta in a Multiparous Woman

**DOI:** 10.1155/2010/329618

**Published:** 2010-09-30

**Authors:** Jennifer C. Hunt

**Affiliations:** Department of Obstetrics, Gynecology, and Reproductive Sciences, University of Manitoba, Women's Hospital, Room WS125, 735 Notre Dame Avenue, Winnipeg, MB, Canada R3E 0L8

## Abstract

Placenta accreta refers to any abnormally invasive placental implantation. Diagnosis is suspected postpartum with failed delivery of a retained placenta. Massive obstetrical hemorrhage is a known complication, often requiring peripartum hysterectomy. We report a case of presumed placenta accreta in a patient following failed manual removal of a retained placenta. We describe an attempt at conservative management with methotrexate in a stable patient desiring future fertility. Treatment was unsuccessful and led to the development of a disseminated intrauterine infection complicated by a bowel obstruction, requiring both a hysterectomy and small bowel resection. In hemodynamically stable patients, conservative management of placenta accreta may involve leaving placental tissue *in situ* with subsequent administration of methotrexate. However, ongoing close observation is required to identify complications.

## 1. Introduction

Placenta accreta is an obstetrical complication associated with significant maternal morbidity and mortality. It is caused by a defect in the decidua basalis resulting in an abnormally invasive placental implantation [1]. This disruption is often related to previous uterine scars, including caesarean sections and prior uterine curettage [2]. Other risk factors associated with placenta accreta are multiparity (>6 pregnancies); placenta previa; prior intrauterine infections; elevated maternal serum alpha-fetoprotein; and maternal age over 35 years [2–4]. Histologically, placenta accreta is identified by trophoblastic invasion of the myometrium in the absence of intervening decidua [1]. The spectrum includes invasion of the superficial myometrium (accreta), invasion into deeper myometrial layers (increta), and invasion through the serosa and/or adjacent pelvic organs (percreta) [5].

Ideally, the diagnosis might be evaluated antenatally in high-risk pregnancies and suspected using ultrasound [1]. This could allow for predelivery planning to reduce maternal morbidity and mortality. Unfortunately, most cases are identified only at the time of delivery when forcible attempts at manual removal of the placenta are unsuccessful [6]. Severe postpartum hemorrhage may result and may lead to complications such as massive transfusion of blood products; DIC; acute renal failure; infectious morbidities; ARDS; loss of fertility [1]. Mortality is as high as 7% [7].

Traditionally, caesarean hysterectomy at the time of delivery has been the preferred management strategy for placenta accreta [1]. Not only does this approach preclude future fertility, but it is also a procedure synonymous with significant perioperative risks [7]. For women who wish to conserve their reproductive function, other treatment options have been described. In some settings, uterine conservation (with the placenta left *in situ*) may be an alternative strategy [5–9]. Adjuvant therapy with methotrexate has also been used to expedite resorption of placental tissue [6–8].

## 2. The Case

A 32-year-old multiparous woman (gravida 7, para 6) presented to a nursing station in Arviat, Nunavut at 34 weeks gestational age in preterm labour. The patient subsequently delivered precipitously prior to transfer to a tertiary care centre. Her obstetrical history was significant for six previous vaginal deliveries and two neonatal deaths secondary to an autosomal recessive metabolic disorder. She had been transferred to a tertiary centre in Winnipeg twice during her current pregnancy for episodes of antepartum hemorrhage. At 19 weeks, she was admitted to hospital for observation and was discharged home after 24 hours. At 32 weeks, she was admitted again for vaginal bleeding. On this admission, her hemoglobin was 78 g/L. A subsequent fetal assessment confirmed an unremarkable right anterolateral placenta that was clear of the internal os and an active fetus with a biophysical profile score of 8/8. No evidence of abruption was noted. She was discharged home.

Following spontaneous vaginal delivery, the placenta failed to deliver over the next eight hours. The patient was transferred to a community hospital in northern Manitoba for manual removal. Upon arrival, she was hemodynamically stable without significant vaginal bleeding. The patient was taken to the operating room to undergo manual removal of the retained placenta. The surgeon was only able to remove small fragments of tissue as the remainder of the placenta was densely adherent to the myometrium. Intraoperative vaginal bleeding occurred and was managed with a combination of vigorous fundal massage, uterine packing, oxytocin, and carboprost. The bleeding settled over the following 18 hours, at which point the packing was removed. She received prophylactic intravenous antibiotics and three units of packed red blood cells. On postpartum day 2, an abdominal ultrasound was performed. A large 12 cm soft tissue mass was visualized at the uterine fundus that was felt to be invading the myometrium. 

**Figure 1:**
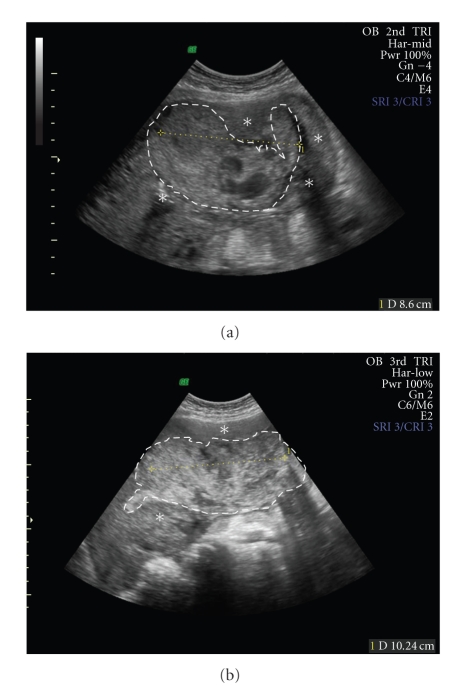
Transabdominal ultrasound image taken on postpartum day 17. This view of the uterine fundus demonstrates significant persistence of retained placental tissue approximately two weeks after methotrexate administration. Serum *β*hCG was 1571 UI/L. The distinction between the placental mass (dotted line) and myometrium (asterisk) is shown. Transabdominal ultrasound image taken on postpartum day 24. This view of the uterine fundus shows significant persistence of retained placental tissue approximately three weeks after methotrexate administration. Serum *β*hCG was 479 UI/L. The distinction between the placental mass (dotted line) and myometrium (asterisk) is depicted.

On postpartum day 3, only mild vaginal bleeding had occurred, and the patient was transferred to a tertiary centre in Winnipeg with a presumed diagnosis of placenta accreta. Upon arrival, her serum *β*hCG was 16 320 IU/L. The following day, she received a single dose of 75 mg of methotrexate intramuscularly and was started on a course of prophylactic cephalexin. Management then continued as an outpatient with twice-weekly visits to clinic to monitor for infection and bleeding. By postpartum day 16, her serum *β*hCG had decreased to 1447 UI/L and her hemoglobin to 79 g/L. Upon assessment in clinic that day, she described ongoing mild-to-moderate vaginal bleeding and was found to have an isolated temperature of 38.5°C. The patient was admitted to hospital for 48 hours. Blood cultures were taken, two units of packed red blood cells were transfused, and oral cephalexin was continued. An abdominal ultrasound (Figure 1(a)) revealed persistent retained placental tissue.

Over the next week, the patient was again followed in an outpatient setting (Figure 1(b)). A repeat *β*hCG was 479 UI/L. On postpartum day 26, she reported a five-day history of nausea, vomiting, reduced oral intake, abdominal pain with eating, and failure to pass stool or flatus. Vaginal bleeding remained unchanged. On examination, she was found to have significant abdominal distension and tenderness on palpation. She was afebrile with a heart rate of 100 beats per minute and blood pressure of 96/58. The patient was admitted for investigation. She had evidence of leukocytosis (white blood cells 21.7 × 10^9^/L), thrombocytosis (platelets 560 × 10^9^/L), and pre-renal failure (creatinine 140 *μ*mol/L, urea 12.6 mmol/L). An abdominal plain film demonstrated dilated loops of bowel and multiple air-fluid levels consistent with a small bowel obstruction. At this time, blood cultures from the previous admission had grown *Prevotella corporis.*

**Figure 2:**
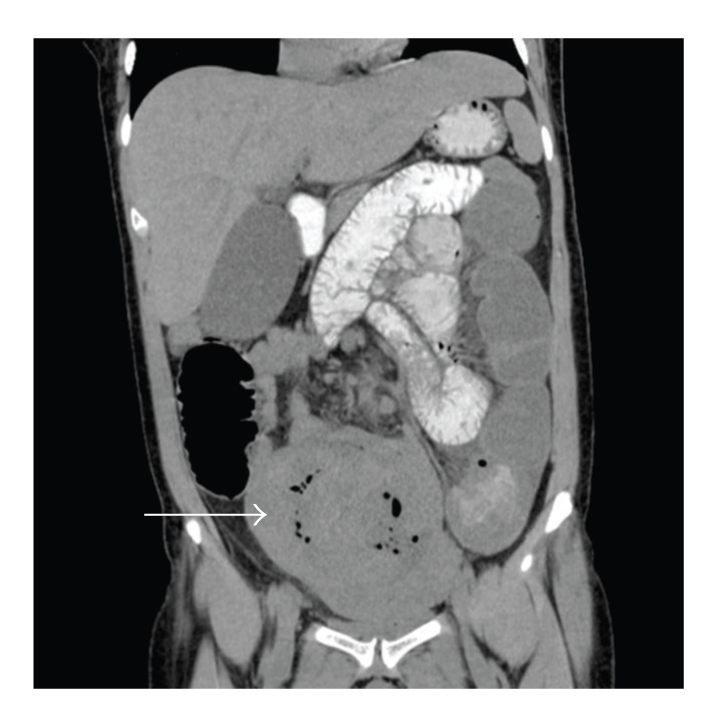
CT scan of the patient's abdomen on postpartum day 27 demonstrating the uterus with the retained placenta was demonstrated (arrow). The presence of dilated, fluid filled loops of small bowel with collapse of the large bowel confirmed the diagnosis of a mechanical small bowel obstruction. A possible adhesion was noted between the uterine fundus and the bowel (not shown here).

The patient was admitted for hydration, bowel rest, pain management, and intravenous antibiotics. Intravenous cefoxitin 1 g every eight hours was chosen based on the blood culture sensitivities. A CT abdomen confirmed a mechanical small bowel obstruction with a possible adhesion noted between the uterine fundus and small bowel (Figure 2). Placental tissue was again noted at the fundus. In consultation with the general surgery service, the obstruction was to be managed conservatively. A consultation was also requested with a senior gynecologist regarding management of the retained placental tissue. The prevailing consensus was that an endomyometritis had occurred secondary to the placenta accreta and had progressed to bacteremia, peritonitis, and intestinal ileus. The patient was consented for an elective dilatation and curettage to be carried out within the next few days. She was received informed consent with respect to possible complications, including laparotomy and hysterectomy. 

On postpartum day 29, the patient was taken to the operating room for a dilatation and curettage. During the procedure, a uterine perforation identified as bowel was pulled through the cervix with forceps. The surgeon proceeded to laparotomy. A large necrotic defect with purulent material was noted at the uterine fundus. Swabs were taken for culture and subsequently grew *Prevotella* species. Furthermore, the small bowel and left ovary were adherent to the uterus. Surgical management of the findings included a total hysterectomy, in addition to an Intraoperative consultation with a general surgeon for a small bowel resection with primary reanastomosis. Blood loss was 100 mL. 

By the evening of postoperative day 0, the patient had become increasingly tachycardic and hypotensive. Urine output decreased dramatically, and significant abdominal distension was found. Serum hemoglobin decreased from 93 g/L to 81 g/L. Postoperative hemoperitoneum was diagnosed. She received volume resuscitation with crystalloid, colloid, and packed red blood cells. The patient returned to the operating room for an emergency exploratory laparotomy. Upon entry, bleeding was noted from the right infundibulopelvic pedicle and left vaginal cuff angle. All bleeding pedicles were reinforced, and good hemostasis was achieved. 

Postoperatively the patient did well. She received further bowel rest with total parenteral nutrition until she was consistently passing flatus and able to tolerate oral intake. A mild wound infection developed on postoperative day 4, and antibiotic therapy was changed to oral amoxicillin/clavulin based on culture sensitivities to cover *Prevotella* (blood), *E. coli* (wound), and non-hemolytic streptococcus (wound). The patient was finally discharged from hospital in good condition on postoperative day 10. Pathology confirmed the presence of necrotic and infarcted villous tissue at the uterine fundus consistent with placenta accreta. Acute and chronic serositis of the small bowel was noted. Incidentally, the cervix was found to have high-grade dysplasia.

## 3. Discussion

The incidence of placenta accreta approximates 1 in 1000 deliveries and has been increasing largely due to the global increase in caesarean deliveries. Patients at risk for abnormal placentation should be assessed antenally by ultrasonography, with or without adjunct magnetic resonance imaging if indicated [1]. The women at the highest risk are those with placenta previa in the current pregnancy and a history of prior caesarean delivery. 

Optimal management of abnormally invasive placentation remains unclear. Traditionally, primary hysterectomy at the time of caesarean section has been the mainstay of therapy particularly in cases where the diagnosis has been discovered antenatally [10]. This procedure has been associated with significant maternal morbidity and mortality. In a recent systematic review, emergency postpartum hysterectomy was found to be associated with maternal morbidity in 56% of cases and with a mortality rate of 3% [11]. In addition to obvious loss of fertility, complications include injury to the gastrointestinal or urinary tracts, infection, as well as massive obstetrical hemorrhage and its sequelae [6, 11]. Furthermore, it has been recognized that planned caesarean hysterectomy is associated with fewer perioperative complications compared to emergent procedures [12]. 

When an extirpative approach is utilized to excise the placenta from the uterus, severe bleeding necessitating urgent hysterectomy may occur [7]. This scenario is encountered when placenta accreta is diagnosed peripartum following failed removal of a retained placenta.

Several reports have described the use of more conservative strategies aimed at preserving the uterus and maintaining future fertility [5–9]. This approach involves leaving the placental tissue *in situ* providing that the patient remains hemodynamic stable, without life-threatening hemorrhage, and with a desire for ongoing fertility. Presuming the diagnosis at placenta accreta can be anticipated, efforts to minimize blood loss may be utilized. These might include having blood products and uterotonic agents available for delivery; blood cell saver technology; compression sutures; uterine packing; selective arterial embolization and/or balloon occlusion; and uterine and/or hypopgastric artery ligation [1]. Although prenatal imaging may be useful, ultrasound findings suggestive of placenta accreta may not always be appreciated. In one review, only 44% of cases were suspected on the basis of antenatal ultrasound [9].

Methotrexate has also been described an as adjuvant therapy for the conservative management of placenta accreta [1, 6–9]. It has been hypothesized that methotrexate acts by inducing placental necrosis and expediting a more rapid involution of the placenta [13]. This contradicts the belief that methotrexate acts only on rapidly dividing cells, given that trophoblast proliferation is not felt to occur at term [14]. Thus, there is controversy as to the effectiveness of methotrexate as an adjuvant treatment. Also, there is a lack of consensus regarding optimal dosing, frequency, or route of administration. In this particular case, a single dose of 50 mg per m^2^ of body surface area was used, similar to the protocol used in the management of ectopic pregnancy at our centre.

In a recent review, conservative management was utilized in 167 cases of placenta accreta/percreta. [9]. The failure rate was 22% and hysterectomy, either primary or delayed, was required mostly for severe hemorrhage. Severe maternal morbidity, including one maternal death, occurred in 6% of cases. The death was attributed to aplasia and nephrotoxicity secondary to intraumbilical administration of methotrexate. This case highlights the adverse effects that may occur following even a single dose of adjuvant methotrexate. 

Another retrospective review of 60 cases of placenta accreta found similar outcomes among women managed conservatively with the placenta *in situ* whether or not adjuvant treatments were used [6]. Of the twenty-six women managed without the use of additional therapies, 22 (85%) had a favourable outcome. In the remaining 4 cases, conservative management failed, and a hysterectomy was required either due to severe hemorrhage or infection. An additional 22 women received methotrexate. Treatment failed in 5 patients; thus, hysterectomy was avoided in 77% of cases. 

Kayem et al. compare two strategies of management in their centre over two different time periods [7]. In the first time period, a policy of removing all placenta tissue was employed (13 cases). In the second, the standard of care changed to leaving the placenta *in situ* (20 cases). The hysterectomy rates in the first and second time periods were 85% and 15%, respectively. Most cases occurred in women where the diagnosis was recognized at the time of delivery and were subsequent to severe hemorrhage or infection.

Although conservative management of placenta accreta appears to be successful at preventing hysterectomy in most cases, there is still potential for morbidity. If such an approach is used, intensive monitoring for complications is required. Women may continue to be at risk for weeks to months after delivery. Sentilhes et al. reported a median period to delayed hysterectomy of 22 weeks [9]. 

Fever is a commonly reported complication. Most cases are secondary to endomyometritis or florid sepsis; however, fever may also represent an inflammatory response to tissue necrosis in the absence of an infectious source [13]. Prophylactic broad-spectrum antibiotic therapy may reduce the incidence of infectious morbidity [6–8]. In our case, the patient developed *Prevotella corporis* bacteremia despite the use of prophylactic antibiotics. Prevotella species are anaerobic gram-negative bacilli that can be implicated in puerperal infections [15]. Therapy must be adjusted based on culture sensitivities as up to one third of strains may be resistant to *β*-lactam antimicrobials [15]. 

Vaginal bleeding may also complicate management for several months following delivery. In Timmermans' review, fever and vaginal bleeding each occurred in 35% of patients and were the inciting factors leading to hysterectomy in all but one (92%) of the patients with treatment failure [6]. Similarly, Sentilhes et al. reported delayed vaginal bleeding as the reason for hysterectomy in 8 of the 36 treatment failures (22%), while sepsis was implicated in 7 cases [9]. Kayem et al. describe a significantly lower occurrence of disseminated intravascular coagulopathy, presumably secondary to severe hemorrhage, in women managed with the placenta in situ (5%) compared to cases where the placental tissue was removed (39%) [7].

Another controversy surrounding the use of methotrexate in the management of placenta accreta has been the utility of monitoring serum *β*hCG. The prognostic implications of decreasing *β*hCG levels following administration of methotrexate are better described in the setting of ectopic pregnancy. For placenta accrete, it is not clear whether decreasing levels correlate with the rate of involution of placental tissue. In one study, the serum *β*hCG levels decreased with a half-life of 5.2 days in women managed by leaving the placenta *in situ* and did not vary with the volume of remaining tissue [16]. Another report described a half-life of serum *β*hCG of 5.8 days in cases of retained placenta managed with methotrexate [17].

## 4. Conclusion

We report a case of placenta accreta in a multiparous patient that was conservatively managed with the placenta *in situ* and adjuvant methotrexate. Although initially the patient did well, she developed severe endomyometritis at the placental site that spread to involve the small bowel, a rare complication not previously reported by other authors. The conservative approach failed as a subsequent hysterectomy, and bowel resection were required. Furthermore, despite the significant decrease in the serum *β*hCG levels, a large placental mass persisted as evidenced by repeat ultrasound examinations during her management. 

This case depicts a common complication associated with this approach to treatment of placenta accreta as well as the need for intensive monitoring. Despite the use of prophylactic antibiotics, our patient developed endomyometritis and bacteremia. Nonetheless, experience in the literature suggests that conservative management of placenta accreta may be valid in select patients with favourable outcomes. 

##  Conflict of Interests

The patient described in this paper has provided written consent for its publication. The author has no conflict of interests to disclose.

## Abbreviations

ARDS: Acute respiratory distress syndrome
*β*hCG: *β*-human chorionic gonadotrophin
DIC: Disseminated intravascular coagulopathy
CT: Computed tomography.
